# China’s Gridded Manufacturing Dataset

**DOI:** 10.1038/s41597-022-01848-8

**Published:** 2022-12-02

**Authors:** Chenjing Fan, Xinran Huang, Lin Zhou, Zhenyu Gai, Chaoyang Zhu, Haole Zhang

**Affiliations:** 1 Research Center for Digital Innovation Design, Nanjing Forestry University, Nanjing, China; 2 College of Landscape Architecture, Nanjing Forestry University, Nanjing, China; 3 School of Public Administration and Policy, Renmin University of China, Beijing, China; 4 Institute of Industrial Economics, Chinese Academy of Social Sciences, Beijing, China; 5 Faculty of Architecture, Civil and Transportation Engineering, Beijing University of Technology, Beijing, China; 6 Shanghai Tongji Urban Planning & Design Institute CO. LTD, Shanghai, China

**Keywords:** Geography, Research data, Industry

## Abstract

The growth of the manufacturing industry is the engine of rapid economic growth in developing regions. Characterizing the geographical distribution of manufacturing firms is critically important for scientists and policymakers. However, data on the manufacturing industry used in previous studies either have a low spatial resolution (or fuzzy classification) or high-resolution information is lacking. Here, we propose a map point-of-interest classification method based on machine learning technology and build a dataset of the distribution of Chinese manufacturing firms called the Gridded Manufacturing Dataset. This dataset includes the number and type of manufacturing firms at a 0.01° latitude by 0.01° longitude scale. It includes all manufacturing firms (classified into seven categories) in China in 2015 (4.56 million) and 2019 (6.19 million). This dataset can be used to characterize temporal and spatial patterns in the distribution of manufacturing firms as well as reveal the mechanisms underlying the development of the manufacturing industry and changes in regional economic policies.

## Abstract (summary)

**Table:** 

Measurement(s)	distribution of manufacturing firms
Technology Type(s)	natural language processing

## Background & Summary

Since the First Industrial Revolution in Britain in the 1760s, manufacturing has been one of the major drivers of the world’s economic development. The center of gravity of the global manufacturing industry has shifted many times due to changes in the world’s economic layout^1^. In the early 1980s, labor-intensive, low-technology, and high-energy industries became less prevalent in the United States, The United Kingdom, Japan, and other developed countries, as well as the “Four Tigers” of East Asia (South Korea, Singapore, Malaysia, Taiwan) and other emerging industrial countries. China, which is the largest developing country in the world, has begun to play a key role in international cooperation in the division of labor and the global manufacturing value chain^2^. Over the next 40 years, the implementation of the reform and opening-up policy has promoted the rapid growth of the manufacturing industry, industrial transformation and upgrading, and the globalization of the economy^3^. The added value of China’s manufacturing sector reached 26.59 trillion yuan, and it has ranked first worldwide for 12 consecutive years, accounting for nearly 30% of the global manufacturing sector and generating more than 100 million jobs.

Regional manufacturing expansion is characterized by both temporal and spatial changes. As the quality and quantity of the manufacturing industry grow over time^4^, the manufacturing industry eventually becomes concentrated in a few regions^5^. This industry is the driving force behind migration, industrial upgrading, segregation, and many other social phenomena^6,7^. China’s coastal areas are densely populated and thus provide an ideal region for the agglomeration of the manufacturing industry; these regions have thus made major contributions to China’s economic growth^8–10^. The rapid increase in land, labor, and other costs, coupled with the implementation of China’s regional development strategies aimed at reducing regional differences, such as the development of the western region and the rise of the central region, the manufacturing industry, especially labor-intensive industries, have begun to move to central and western regions on a large scale^11,12^.

Few empirical studies of Chinese manufacturing firms have been conducted at the firm level, as detailed geographic data on various industrial firms are often not readily available (Table 1). Most research on the distribution of industry has been conducted at the macro provincial and municipal levels^13,14^; changes in the spatial pattern of the manufacturing industry often need to be analyzed using more micro-scale data. However, obtaining official micro-scale data is often difficult either because access to some data is prohibited by certain policies or because the data have never been collected and processed. Table 1 shows a comparison of existing data collection methods for studying industrial patterns. As these data collection methods often lack classification systems or accurate geographic data, they have various limitations, which results in poor quality data that are lacking in temporal and spatial resolution. The most accessible databases are from the Industrial Enterprises Database (IED)^6,15^; however, these data have only been taken at large scales and at a firm’s registered address, which can often differ from the address at which the operations of the firm are carried out. For example, records of small-scale branch factories are lacking^6^. More detailed quantitative data would greatly improve the robustness of industry-related research.

**Table 1:** Comparison of data collection methods used in current research examining industrial patterns.

Data Source	Author	Region	Data Source Reliab ility	Sample Size	Classification System	Accuracy of Geographic Data	Temporal Resolution	Accessibility
Statistical Yearbook	(Qiliang Mao, 2014)^8^	Provinces in China	✶	✶	×	▲ District Statistics	✶	✶
	(Wenbin Zhang, 2007)^37^	Provinces in China						
	(Ruibo Zhou, 2017)^38^	Guangdong, China						
Industrial Enterprise Database (IED)	(Jie Zhang, 2018)^39^	Zhejiang, China	✶	▲	✶	▲ Address	▲	✶
	(Xiaoping Zhang, 2012)^40^	Beijing, China						
	(Junsong Wang, 2014)^41^	The Yangtze River Delta, China						
Economic Census Data	(Xiaohan Yin, 2019)^42^	Beijing, China	✶	✶	×	×	▲	×
	(Xiaoye Chen, 2017)^43^	Shanghai, China						
	(Rachel Guillain, 2010)^44^	Paris, France						
Questionnaire	(Wenhui Cao, 2016)^45^	Jiangsu, China	▲	×	✶	✶ Latitude and Longitude	✶	✶
	(Tongliang An, 2006)^46^	Jiangsu, China						
	(Ilan Elgar, 2010)^47^	Toronto, Canada						
Business Registration Data	(Lei Dong, 2021)^6^	Provinces in China	✶	▲	✶	▲ Address	✶	▲
	(Fa Li, 2018)^15^	Cities in China						
Night Light Data	(Xi Chen, 2011)^48^	The US	▲	×	×	×	✶	✶
	(J. Vernon Henderson, 2012)^49^	Countries in the World						
Street View Images	(Nikhil Naik, 2017)^50^	Communities in the US	▲	×	×	✶ Latitude and Longitude	×	✶
Socioeconomic Datasets	(Charlotta Mellander, 2015)^51^	Cities in Sweden	✶	✶	×	×	✶	✶
Map POI (Point of Interest)	(Bing Xue, 2020)^19^	Shenyang, China	✶	✶	▲ Built-in Map or Manual Classification	✶ Latitude and Longitude	✶	✶
	(Sergio Porta, 2009)^52^	Bologna, Italy						
Land Transfer Data	(Lin Zhou, 2019)^53^	Prefecture-level cities in China	✶	✶	✶	▲ Address	✶	▲
Small Scope Permit Data Provided by the Government	(Hongwei Dong, 2013)^54^	Metropolitan Areas in Portland	✶	▲	▲	▲ Address	▲	▲
Cellphone Data	(Lei Dong, 2017)^55^	Beijing, China	Unavailable					
	(Angela Airoldi, 2006)^56^	Milan, Italy						
Social Media	(Alejandro Llorente, 2015)^57^	Cities in Spain	Unavailable					
	(Yongqiang Lv, 2021)^58^	Cities in China						

Note: ✶ indicates good, ▲ indicates general, × indicates none.

**Table 2:** The relationship between industries in the IED and the manufacturing 155 classification in this paper.

Classification of the manufacturing industry in the GMD in this paper	Industry
Textile and garment	Textile industry Textile clothing, apparel industry Leather, fur, feather, and their products and footwear
Mechatronics and equipment	Electrical machinery and equipment manufacturing General equipment manufacturing Special equipment manufacturing Manufacturing of computers, communications, and other electronic equipment Automobile industry Manufacturing of railway, marine, aerospace, and other transportation equipment Instrument manufacturing Repair of metal products, machinery, and equipment
Wood furniture	Wood processing and wood, bamboo, rattan, palm, grass products Furniture manufacturing
Agricultural and sideline products food processing	Agricultural and sideline food processing industry Food manufacturing Wine, beverage, and refined tea manufacturing Tobacco products industry
Metallurgical chemical industry and resource rough processing	Chemical raw materials and chemical products manufacturing Metal products industry Rubber and plastic products Ferrous metal smelting and rolling processing industry Nonferrous metal smelting and rolling processing industry Petroleum processing, coking, and nuclear fuel processing industries Chemical fiber manufacturing Comprehensive utilization of waste resources Nonmetallic mineral products industry
Papermaking culture	Culture and education, art, sports, and recreation goods manufacturing Paper and paper products industry Printing and recording media reproduction
Pharmaceutical manufacturing	Pharmaceutical manufacturing
Other manufacturing	Other manufacturing
Non-manufacturing in secondary industry and other non-manufacturing industries	Mining, electricity, heat, gas, and water production & supply

The distribution of classified manufacturing firms could be mapped by combining the strengths of the manufacturing classification of the IED with the strengths of map POI data. The IED is the most promising source of data for mapping the distribution of classified manufacturing firms, as it provides accurate firm names and manufacturing classifications (Table 1). However, these data are only sample data and lack latitude and longitude coordinates for the firms, and the statistical objects are large and include medium-sized manufacturing firms with an annual turnover of more than 20 million yuan in China, which means that the original IED does not include manufacturing firms with an annual turnover of less than 20 million yuan^16,17^. Map POI data have precise spatial information and have been widely used in various fields^18^. One of the greatest challenges in manufacturing research is the absence of a clear classification system of manufacturing firms, yet a robust manufacturing classification system is essential if map POI data are used to study the distribution of manufacturing firms. However, the manual processing of data is time and labor-consuming^14,19–23^. The machine learning method, coupled with small sample classification data, such as the “Firm name – Manufacturing classification” in the IED, can be used to identify manufacturing types in the map POI data and develop base maps for manufacturing research.

## Methods

**Fig. 1:**
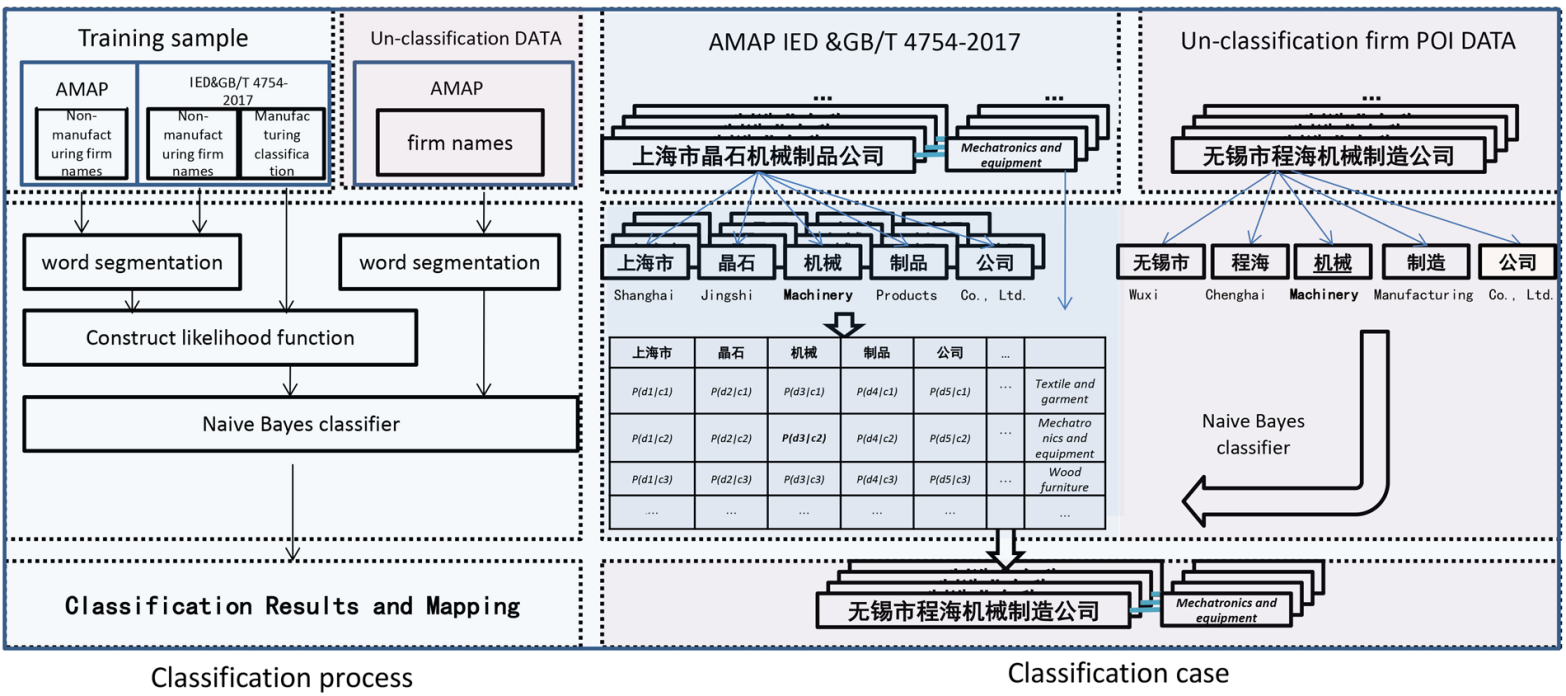
Research framework and classification example.

The number of POI is nearly equal to the actual number of facilities in cities^18^. Therefore, large-scale manufacturing patterns can be studied at high spatial resolution using the POI classification. In this paper, seven types of Chinese gridded manufacturing datasets (GMDs) were generated, which used IED data as accurate training sample data to classify and identify map POIs through machine learning. Data on the distribution of seven categories of manufacturing in China were obtained. The technical process employed includes four steps:

- Building a learning sample library for manufacturing types by the IED.
- Collecting and pre-processing map POI data^24^.
- Classifying manufacturing based on machine learning.
- Drawing the high spatial resolution grid map of China’s manufacturing industry in 2015 and 2019 (Fig. 1).

After the data were produced, their quality was tested.

### Construction of the “name-manufacturing type” machine learning sample database using IED

The name-manufacturing type learning sample database was constructed using IED. According to the naming method of Chinese firms and Fa Li’s (2018) approach, there is a link between the name of a facility and the type of manufacturing industry to which it belongs^15^. Thus, a name-manufacturing sample database or keyword dataset can be constructed for machine learning to study the manufacturing classification. In the process of searching for relevant literature, we found that the IED can be used as the learning sample database. China’s IED, established by the National Bureau of Statistics, covers all state-owned industrial firms and non-state-owned secondary sector firms above a designated size, with manufacturing accounting for more than 90% of the statistics. The IED data sources are official, but they only sample large and medium-sized manufacturing firms with an annual turnover of more than 20 million yuan in China and lack geographic coordinates and branch factory addresses. Data from the IED for China in 2013 include some large firms (ca.  344,875 firms); these have been strictly classified and marked, and their industry names are in strict accordance with the national economic industry classification standards in GB/T 4754-2017. Therefore, machine learning and non-subjective supervised classification can achieve better results than manual labeling using the two-field name - manufacturing type classification in the IED. To reduce the redundancy of classification types, industries with similar types were merged into larger types following the method of Shen (2021)^6,18^. First, we manually marked 27,689 non-manufacturing samples (e.g., service industry, agriculture, and supply industry) as non-manufacturing samples in the classification sample. Next, the names of leading industry types in the development zones in various regions in the 2018 edition of “China Development Zone Review Announcement Catalogue”^25^ were summarized, and the names of different industry types with high frequency were extracted; industry types with similar names were merged into a larger category. The names of the manufacturing industry categories corresponding to the firm names in the IED were then summarized. Finally, the corresponding manufacturing industry was divided into seven categories by combining the development zone and the IED summary classification, which provided the learning sample database classification standard. Therefore, the “Name-Manufacturing Type” machine learning sample database contains 372,564 firm names and their corresponding classification. The study samples are provided at 10.6084/m9.figshare.19808407^26^.

### Collection and preprocessing of map POI data

Nearly every company, enterprise, firm, site, or facility in China (even those not registered with the Industry and Commerce Department) has a specific location on government-approved web maps. Our data were obtained from the Amap website (https://www.amap.com/) through web scraping. Because some factories or firms do not appear on the map after they have gone bankrupt or changed addresses, and remote branch factories are shown on the map, these data have higher precision than questionnaire and statistical data. We collected data using the services provided by Amap’s API and divided them into batches, which involves using web crawlers to collect the POI names and locations of firms across China from January 2nd to 20th, 2015, and from December 20th to 30th, 2019^27,28^. To reduce the potential interference from the original data, preprocessing was carried out as follows:

- “Scenery,” “shopping,” “catering,” “road name,” and other types of built-in classification from the map unrelated to firms were filtered out, and only the firm and its corresponding latitude and longitude were retained to increase the classification speed.
- By identifying and matching word items, points with the same place name but different marks within 1 km around the POI points (e.g., East Entrance, West Gate) were deleted to solve the problem associated with counting the same POI.

The final map POI dataset contained firm names and spatial information (approximately 5.24 million in 2015 and 7.35 million in 2019).

### Map POI name-manufacturing type classification algorithm based on the Naive Bayes algorithm for machine learning

To enable the computer to learn and classify the manufacturing industry samples, Chinese word segmentation was performed on the classified samples and POI names. To transform text into a data structure that a computer can process, the text needs to be sliced into semantic units. In the first step of our machine learning, we used the *jieba* module (the Chinese word segmentation module in the Python) to segment the names of Chinese firms^29,30^. The Chinese Thesaurus was used to perform forward maximum matching for POI name field information, which was segmented into several words for keyword recognition, manual tagging, or machine learning training. Next, meaningless fields for information classification and possible special symbols were removed (such as punctuation, spaces, and bom characters), which will affect the classification results, and only Chinese and English characters were retained.

After word segmentation was complete, a machine learning classifier was built. The Naive Bayes algorithm was used to learn the name-classification samples of the IED after word segmentation. As proposed by F. Sebastiani, text classification can be understood as a function of acquisition, where *S* = {*s*_1_*,s*_2_*, …,s*_*n*_} indicates the document or string to be classified, and *C* = {*c*_1_*,c*_*2*_*… c*_*m*_} represents the set of categories in the predefined classification system. The goal of text classification is to find an evaluable mapping: *f*: S ↦ C.

The classification space is an m-dimensional Euclidean space, and the value of each dimension is in [0,1]; this represents the probability of the input s in each dimension after f mapping, which can be determined by calculating the probability distribution of the category to which s belongs. The formal mathematical definition of text classification is shown in Eq. 1:

1 $${C}_{ij}=\left\{\begin{array}{l}1\;String\;{S}_{i}\;{\rm{belongs}}\;{\rm{to}}\;{\rm{category}}\;j\\ 0\;String\;{S}_{i}\;{\rm{does}}\;{\rm{not}}\;{\rm{belong}}\;{\rm{to}}\;{\rm{category}}\;j\end{array}\right.$$

The Naive Bayes classifier was used in this study. First, a probabilistic evaluation learning approach was used for each matched manufacturing classification in the IED name *S*:

According to the Bayesian Equation, the probability that s belongs to *c*_*i*_ for any input *s* is:

2 $$p({c}_{i}| s)=\frac{p(s| {c}_{i})p({c}_{i})}{p(s)}$$

Equation (2) is the likelihood function of the Bayesian classifier. Maximizing it over *c*_*i*_ gives the class to which the input *s* belongs:

3 $$\bar{c}=argma{x}_{j}{\rm{p}}({c}_{i}| {\rm{s}})$$

That is, $\bar{c}$ is the category that makes the conditional probability *p*(*c*_*i*_|*s*) take the maximum value among all categories *C* = {*c*_*1*_*,c*_*2*_*… c*_*m*_}.

In Eq. (2), *p*(*s*|*c*_*i*_) is the prior probability. To calculate the likelihood function, each prior probability needs to be calculated *p*(*s*|*c*_*i*_).

If we denote the s participle as *s* = {*w*_*1*_*, w*_*2*,_*…, w*_*k*_} and assume that the occurrence probability of each word in *s* is independent of each other, then:

4 $$p\left(s| {c}_{i}\right)=p\left({w}_{1}| {c}_{i}\right)p\left({w}_{2}| {c}_{i}\right)...p\left({w}_{k}| {c}_{i}\right)={\prod }_{i=1}^{k}p\left({w}_{i}| {c}_{i}\right)$$

*p*(*w*_1_|*c*_*i*_) represents the probability that the participle *w*_*i*_ appears in the category *c*_*j*_, and it can be calculated by the following equation:

5 $$\widehat{P}({w}_{i}| c)=\frac{count({w}_{i}c)}{{\sum }_{w\in v}count(w,c)}$$

From Eqs. (2–5), the category to which s belongs can be determined.

We implemented the above word segmentation, learning, and classification process in the Python environment. Based on the text algorithm of the Naive Bayes classification, the IED in 2.1 was used as the machine learning training sample, and map POI data in 2.2 were used as the sample for classification in the above machine learning model; the trained model was applied to the name classification of manufacturing firms. The classification results of POI firm names were imported into the geographic information database combined with the original geographic coordinates of the POI. The model algorithm code is provided in the Figshare repository (10.6084/m9.figshare.19808407)^26^.

### Patterns in China’s manufacturing industry in 2015 and 2019

Scale can substantially affect the results of analyses of spatial economic patterns. If an analysis is conducted at an excessively large-scale, small-scale patterns can be overlooked; by contrast, if the scale of the analysis is too small, general patterns are often not detectable^18^. To fit commonly used LandScan population data (https://landscan.ornl.gov/) and GDP grid data, we divided China into a grid of 0.01° latitude by 0.01° longitude. After classification, seven different types of manufacturing distribution data were projected onto the grid, and the number of points of the different types of manufacturing categories in each grid was counted. Greater numbers of points indicate greater numbers of industries. After data processing, there were 4.56 million (2015) and 6.19 million (2019) firm points.

For this dataset, we first constructed a list of cities with the full names and abbreviations of prefecture-level and above cities in China (including municipalities directly under the Central Government, prefecture-level cities, regions, leagues, autonomous prefectures, Hong Kong, Macao, and Taiwan). More information on the administrative divisions in China is provided in ref. ^31^. In the property table, each grid corresponds to a field, which indicates the province and city to which it belongs and the quantity of each of the seven types of manufacturing industries in each grid in 2015 and 2019. All coordinates were based on the WGS84 projection, and the grid was divided according to LandScan population data (0.01° latitude by 0.01° longitude).

**Fig. 2:**
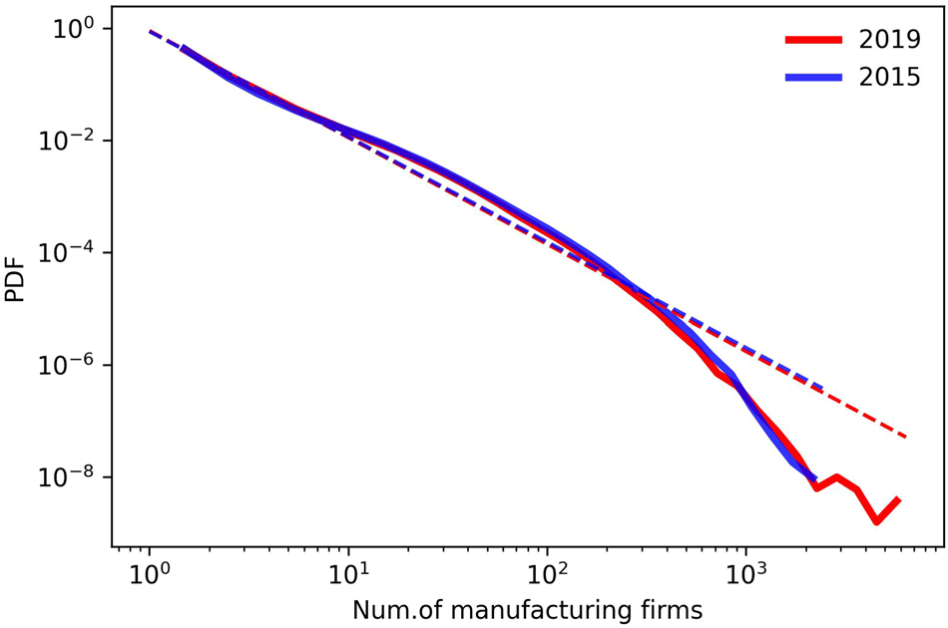
Probability density function of grid data values.

**Fig. 3:**
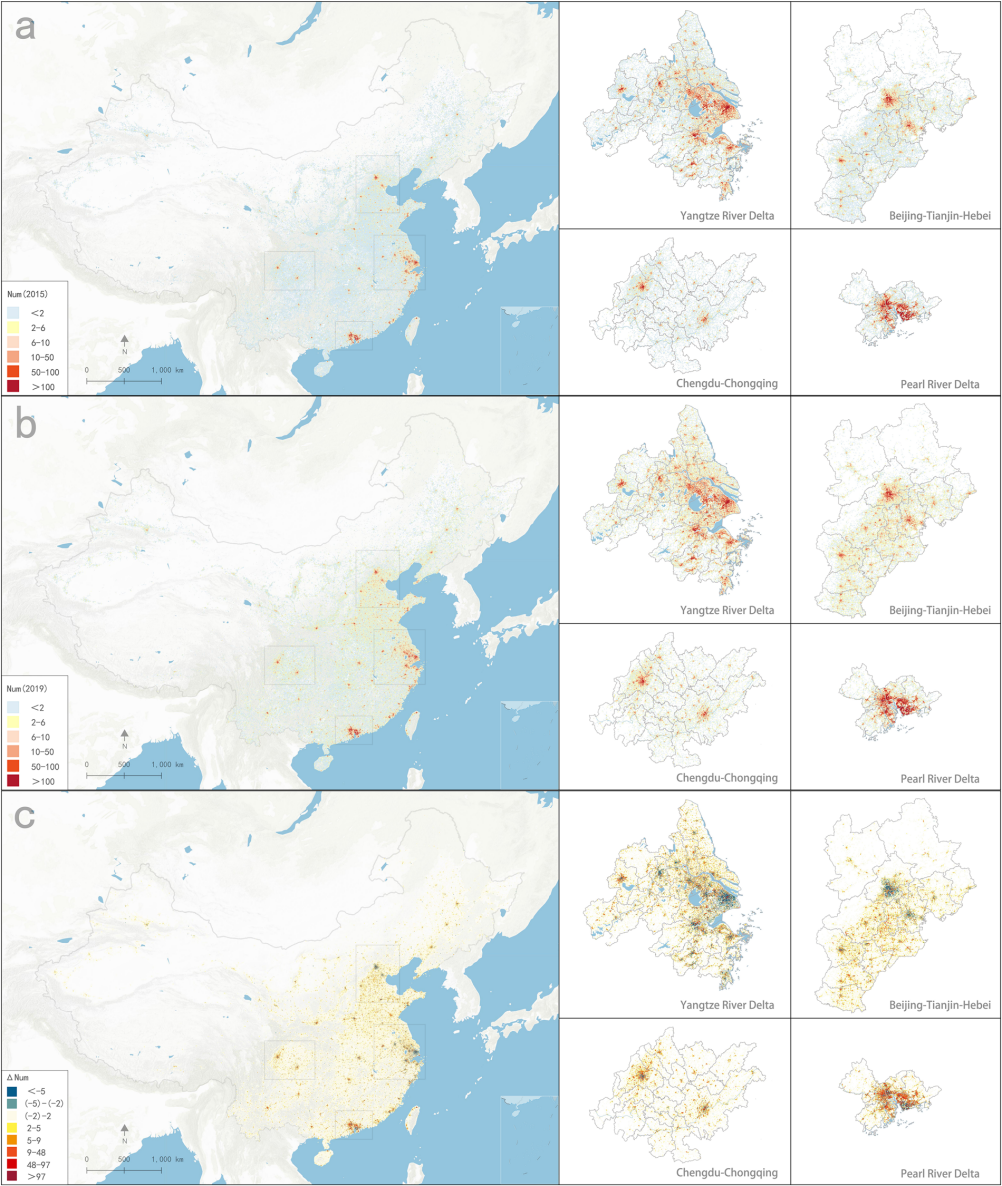
Distribution of China’s manufacturing industries. (**a**) Distribution of manufacturing firms across the whole country, the BTH, the YRD, the PRD, and the CC in 2015. (**b**) Distribution of manufacturing firms across the whole country, the BTH, the YRD, the PRD, and the CC in 2019. (**c**) Increase in the number of manufacturing firms across the whole country, the BTH, the YRD, the PRD, and the CC from 2015 to 2019.

**Fig. 4:**
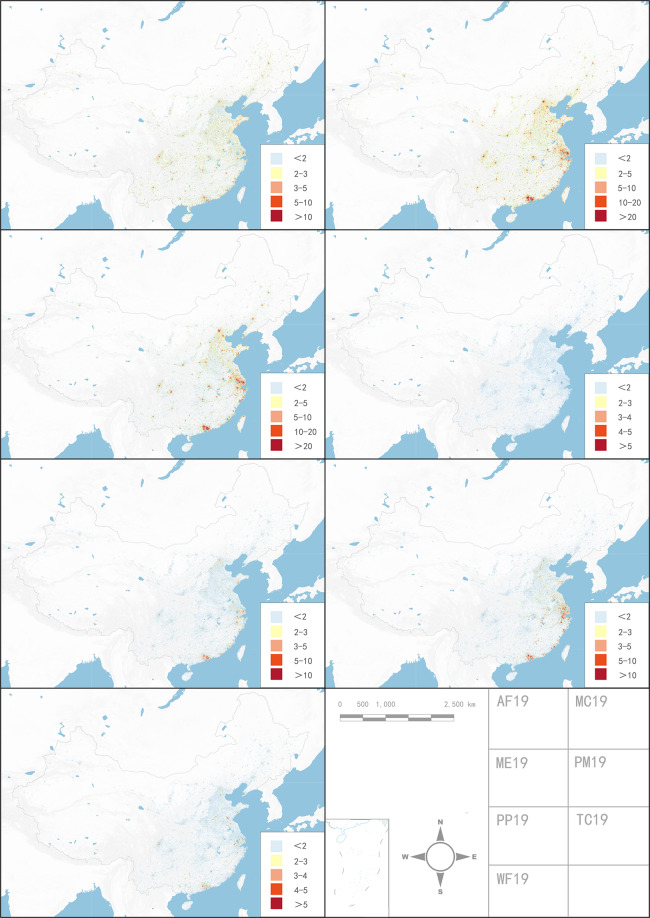
Distribution of different types of manufacturing firms in China.

We found that the distribution of grid values in 2015 and 2019 fit the probability density function well (Fig. 2), which is consistent with the power-law distribution observed for most of the socio-economic characteristics of the large-scale data^32,33^. The geographical changes in China’s manufacturing industry can also be observed in the map (Fig. 3a–c), and most economic activities were concentrated in the eastern coastal areas, especially in the Beijing-Tianjin-Hebei urban agglomeration (BTH), the Yangtze River Delta (YRD), the Pearl River Delta (PRD), and the Chengdu-Chongqing City Group (CC)^11^. Unlike the yearbook data, the changes in manufacturing firm types and their spatial distribution among these four urban agglomerations and inner cities can be clearly observed (Fig. 3 and Fig. 4).

## Data Records

Our data have been deposited in the Figshare repository (10.6084/m9.figshare.19808407)^26^. The database contains an SHP file (data 2015–2019.shp) with field names as shown in Table 3. Each line of the file represents a grid cell record.

**Table 3:** Introduction to the data format.

OBJECTID	Grid cell number
Sum15, Sum19	Number of manufacturing firms in 2015 and 2019
AF15, AF19	Number of food processing firms of agricultural and sideline products in 2015and 2019
MC15, MC19	Number of metallurgical, chemical, and resource rough processing firms in 2015 and 2019
ME15, ME19	Number of mechatronics and equipment firms in 2015 and 2019
OM15, OM19	Number of other manufacturing firms in 2015 and 2019
PM15, PM19	Number of pharmaceutical manufacturing firms in 2015 and 2019
PP15, PP19	Number of papermaking culture firms in 2015 and 2019
TC15, TC19	Number of textile and garment firms in 2015 and 2019
WF15, WF19	Number of wood furniture firms in 2015 and 2019
province	The name of the province where the grid cell is located
city	The name of the prefecture-level city where the grid cell is located

For ease of use, we also included county, city, and province information for each set of cell coordinates. A summary of the basic statistics of the manufacturing industry at the provincial level is shown in Table 4. The number of manufacturing firms is highest in Guangdong, Jiangsu, Shandong, Zhejiang, and Shanghai; thus, these regions have the most developed manufacturing industries.

**Table 4:** Statistical summary of the provincial manufacturing firms in 2015 and 2019.

ID	Province	TC15	ME15	WF15	AF15	OM15	MC15	PM15	PP15	TC19	ME19	WF19	AF19	OM19	MC19	PM19	PP19	SUM15	MAX15	MEAN15	SUM19	MAX19	MEAN19
1	Anhui	8444	40462	2340	13658	80	50200	965	4011	14766	59022	4357	27455	77	83636	1441	6282	120160	402	0.63	197036	505	1.03
2	Beijing	5130	77472	1879	9192	3	51945	716	4791	4513	79507	1519	10875	5	56174	880	4968	151128	953	6.01	158441	792	6.30
3	Chongqing	1883	27122	1111	6028	3	23586	328	1759	2514	37727	1823	11241	4	36606	471	2547	61820	1005	0.56	92933	827	0.84
4	Fujian	22884	46707	3196	18749	73	63133	307	9480	26304	60662	4640	28888	75	85234	411	11763	164529	491	1.03	217977	502	1.36
5	Gansu	373	5397	285	4497	1	11939	338	759	564	7391	471	7397	1	19219	491	1272	23589	157	0.04	36806	204	0.06
6	Guangdong	123573	385959	19897	34640	278	353456	1952	54919	131106	484672	24699	54452	295	446729	2521	63116	974674	1127	4.23	1207590	5169	5.24
7	Guangxi	2251	17781	2258	12410	3	32598	543	3097	3545	26998	5597	22427	10	57119	740	4862	70941	281	0.24	121298	295	0.40
8	Guizhou	641	6950	673	5605	2	17385	314	1053	1267	12297	1960	13805	4	36145	519	2052	32623	346	0.15	68049	257	0.30
9	Hainan	185	2795	246	3112	0	5543	296	404	280	4342	456	5273	0	9367	322	627	12581	433	0.33	20667	318	0.54
10	Hebei	11804	44369	3463	12523	14	74068	958	6043	18794	80225	5605	24849	39	130104	1366	10249	153242	253	0.53	271231	295	0.93
11	Heilongjiang	882	11800	1288	9748	0	17603	435	1299	1127	15509	1800	15690	1	25216	597	1828	43055	228	0.06	61768	169	0.08
12	Henan	6515	43740	2331	16953	13	65307	842	4463	11662	70899	4775	33402	21	113567	1372	7516	140164	423	0.60	243214	342	1.04
13	HongKong	1838	4974	77	1224	3	23368	95	1187	4042	10477	185	2338	4	38428	293	2507	32766	1895	32.64	58274	4011	58.17
14	Hubei	6884	41690	1590	12789	48	47484	861	3261	8584	53272	2504	21292	50	67076	1049	4381	114607	535	0.45	158208	372	0.63
15	Hunan	3686	29811	1704	12469	13	36473	500	3379	4754	44867	3602	23325	17	61783	652	5242	88035	494	0.32	144242	525	0.52
16	InnerMongolia	822	5381	458	5556	0	12831	205	773	1114	8742	743	9630	1	21042	300	1220	26026	84	0.01	42792	175	0.02
17	Jiangsu	69932	249322	8368	22217	94	197002	966	15406	80126	300139	11583	34906	94	250818	1114	17967	563307	705	3.92	696747	1145	4.85
18	Jiangxi	10003	19123	2472	8161	4	35822	564	2927	13591	28925	4844	13680	18	58650	780	4130	79076	213	0.36	124618	175	0.57
19	Jilin	698	12030	973	6829	0	14417	647	995	879	16248	1360	11054	0	22098	856	1431	36589	156	0.12	53926	244	0.18
20	Liaoning	4070	35861	1698	9556	3	37906	450	2244	5013	45249	2619	15499	8	54946	602	2953	91788	605	0.41	126889	544	0.57
21	Macao	39	135	3	24	0	594	13	26	217	380	11	132	0	894	29	64	834	264	53.88	1727	300	112.00
22	Ningxia	330	3021	161	2359	0	8674	84	387	402	4332	231	3818	0	13291	102	559	15016	162	0.21	22735	288	0.31
23	Qinghai	103	1088	50	1038	0	3127	73	240	164	1601	94	1609	0	4858	112	390	5719	50	0.01	8828	97	0.01
24	Shaanxi	1006	23595	767	6774	0	25993	545	1707	1613	33672	1414	13059	4	43484	779	2933	60387	549	0.21	96958	518	0.33
25	Shandong	20304	95260	6908	31445	11	128322	1115	11669	27566	143488	14361	56507	20	206381	1624	16075	295034	383	1.29	466022	397	2.03
26	Shanghai	14655	118980	5041	10767	26	115807	651	10237	12146	117291	3845	11319	18	110573	780	9503	276164	568	29.98	265475	712	28.84
27	Shanxi	607	9510	468	4176	0	18315	388	1039	971	15422	852	7824	3	31896	596	1662	34503	189	0.15	59226	215	0.26
28	Sichuan	4893	45958	3557	17037	8	57502	1325	4705	6252	65387	5768	31748	18	92926	1798	7313	134985	610	0.20	211210	786	0.31
29	Taiwan	351	32247	29	850	0	17002	1	1022	1838	43304	506	4562	0	38557	217	2882	51502	438	1.13	91866	544	2.01
30	Tianjin	1749	26142	915	3499	2	29724	206	2103	1930	30607	1038	4628	4	35826	259	2561	64340	640	3.79	76853	721	4.52
31	Tibet	33	344	30	458	0	1061	61	95	65	719	86	769	0	2298	108	197	2082	32	0.00	4242	43	0.00
32	Xinjiang	853	4982	264	4599	0	13215	113	759	1365	6782	429	7126	1	19625	165	1083	24785	193	0.01	36576	150	0.01
33	Yunnan	785	10867	1071	10045	1	28104	618	2162	1289	17617	2330	20599	2	51609	962	3753	53653	273	0.11	98161	429	0.20
34	Zhejiang	115062	199510	8189	20524	249	175884	792	35812	124878	236130	10857	28478	324	208586	962	37829	556022	819	3.98	648044	971	4.64

(See Table 2 for the names of the manufacturing industries; the sum is the total number of provincial manufacturing firms in the grid; Max and Mean are the maximum and average statistical indicators of the grid, respectively).

## Technical Validation

As quantitative data of the manufacturing industry have not yet been classified, we determined whether the data and their classification were accurate to evaluate the reliability of the data. Technical verification of the data was carried out using three approaches: classification data accuracy verification, grid data verification, and social and economic data verification. Because the official manufacturing distribution data have not been published, and the above data do not represent the actual distribution of manufacturing firms, we provide the classification accuracy for reference after each verification.

### Classification data verification

The purpose of classification data verification is to verify the classification accuracy of the classifier. According to machine learning, the result can be displayed after computing the accessory model algorithm code. The precision index of the training samples was AF = 0.77, MC = 0.92, ME = 0.96, OM = 0.90, PM = 0.82, PP = 0.91, TC = 1.00, and WF = 0.96; the precision of the non-manufacturing class was 0.93. We manually checked 73,500 (≈1% of the total) firm names in 2019, and the total accuracy was 92%. The precision index for each class was AF = 0.86, MC = 0.94, ME = 0.93, OM = 1.00, PM = 0.98, PP = 0.86, TC = 0.97, and WF = 0.99; the precision index for the non-manufacturing class was 0.95.

### Verification with the published gridded data

**Fig. 5:**
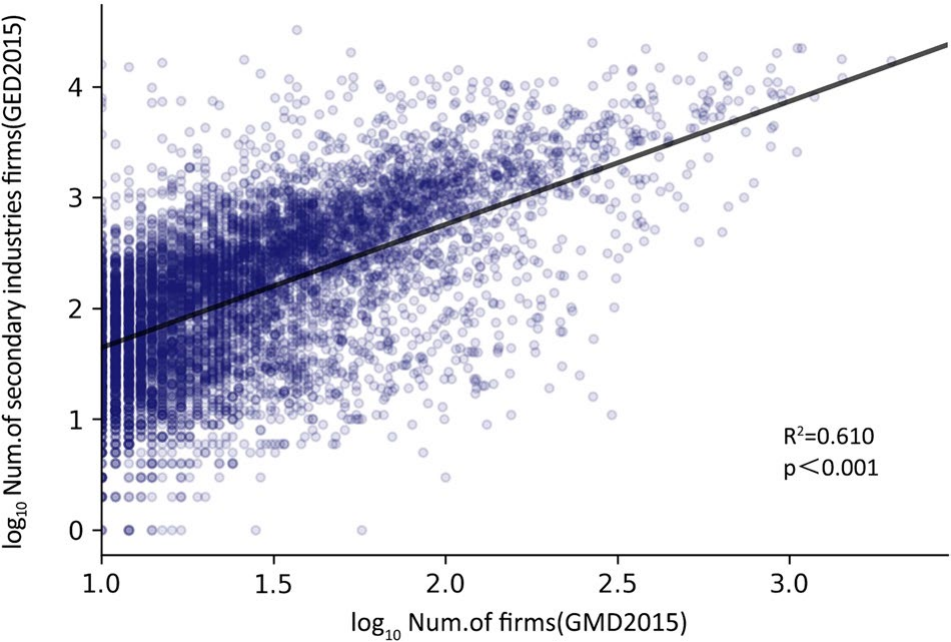
Estimated correlation between the GMD and the GED secondary industries of the manufacturing grid in 2015.

The purpose of the validation with the published gridded establishment dataset (GED) was to determine whether the general patterns in the distribution of the manufacturing firms were accurate. To verify their accuracy, the GED, the only grid data measuring economic activities in mainland China, was obtained from ref. ^6^. We matched our grid data with secondary industry data from the 2015GED and then ran a simple regression to estimate the correlation between the two datasets. The *R*^2^ was 0.620, indicating that the two datasets were consistent (Fig. 5). Some deviation might be explained by the fact that the database comprises data on all the secondary industries in mainland China, whereas our data were on the manufacturing industries across China.

### Verification with social and economic data

The purpose of the validation with social and economic data was to determine (1) industry registration data validation, (2) whether the data describing macroeconomic industries in the yearbook were relevant to the GMD, and (3) whether the relative proportions of the different manufacturing firms were similar to samples from the IED.

Although the above data do not represent the actual distribution of different manufacturing industries, we provided the classification accuracy for reference after each verification.

1. Industry Registration Data Validation
  To verify whether the number of our manufacturing firms is similar to the firm registration data, we aggregated the number of manufacturing firms registered on the Chinese mainland in 2019 from the Qichacha website (https://www.qcc.com/, screened until 2019.12.30), and we compared these data with our 2019 GMD industrial enterprise database. The results of the fitted model are shown in Fig. 6. In general, the total number of manufacturing firms in each city in our data was highly consistent with the number of registered firms in Qichacha (R^2^ of 0.935). The possible reasons for the error include 1) the actual address of the firm is not at the place of registration, and 2) some firms only have registration information but no actual factory address.

**Fig. 6:**
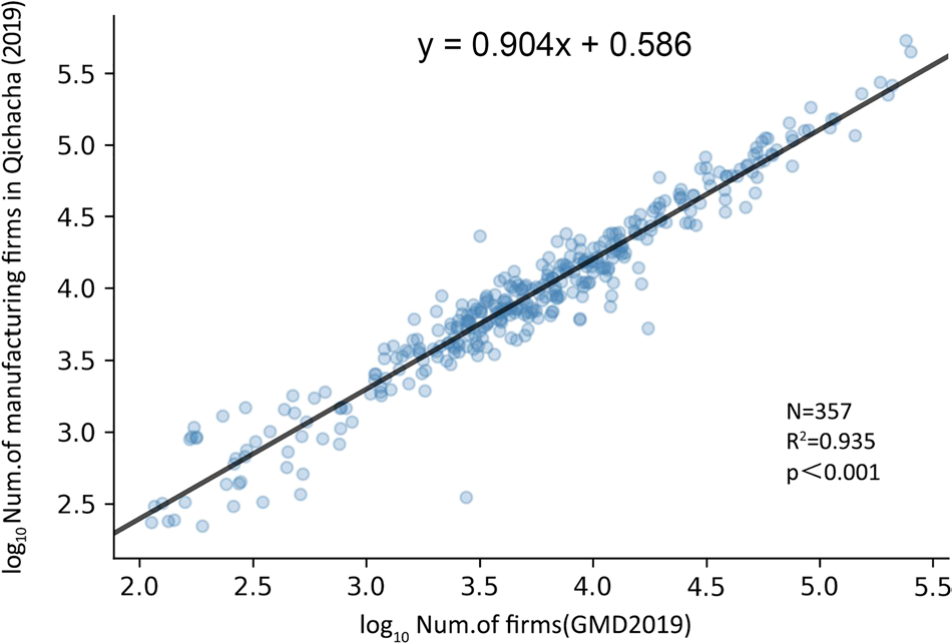
Correlation between the number of manufacturing firms in prefecture-level cities from GMD 2019 and the number of registered firms.
2. Yearbook data verification
  We summarized the number of manufacturing firms at the city level and compared them with the social and economic indicators of secondary GDP and manufacturing employment at the city level. Secondary GDP was derived from the City Statistical Yearbook, and manufacturing employment was derived from the CEIC (China Entrepreneur Investment Club: China Economic Database). Some cities were excluded due to a lack of statistical data. In Figs. 7 and 8, we show the results of the two models: the linear regression of the total number of manufacturing industries with secondary GDP and manufacturing employment. Overall, our data perform well in estimating these socioeconomic variables, with *R*^2^ values exceeding 0.72 in all cases.

**Fig. 7:**
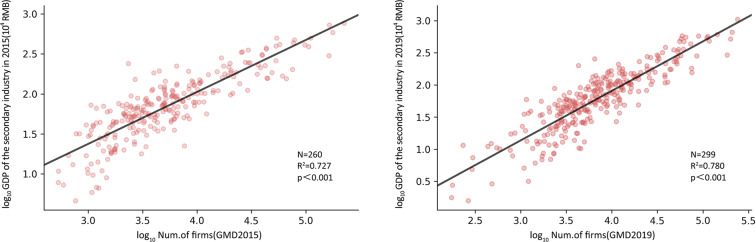
Correlation estimation and verification of the total manufacturing volume of prefecture-level cities and secondary production GDP (a 2015, b 2019).

**Fig. 8:**
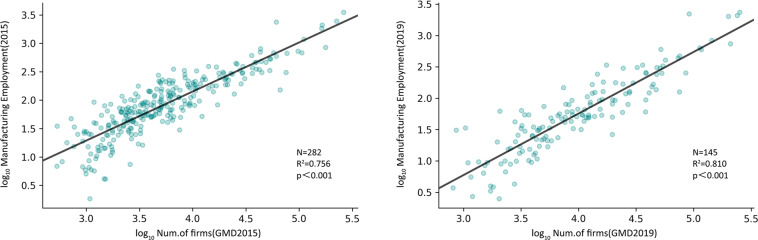
Correlation estimation and verification of the total manufacturing volume of prefecture-level cities and the number of employees in the manufacturing industry (a 2015, b 2019).
3. Manufacturing type classification verification

**Fig. 9:**
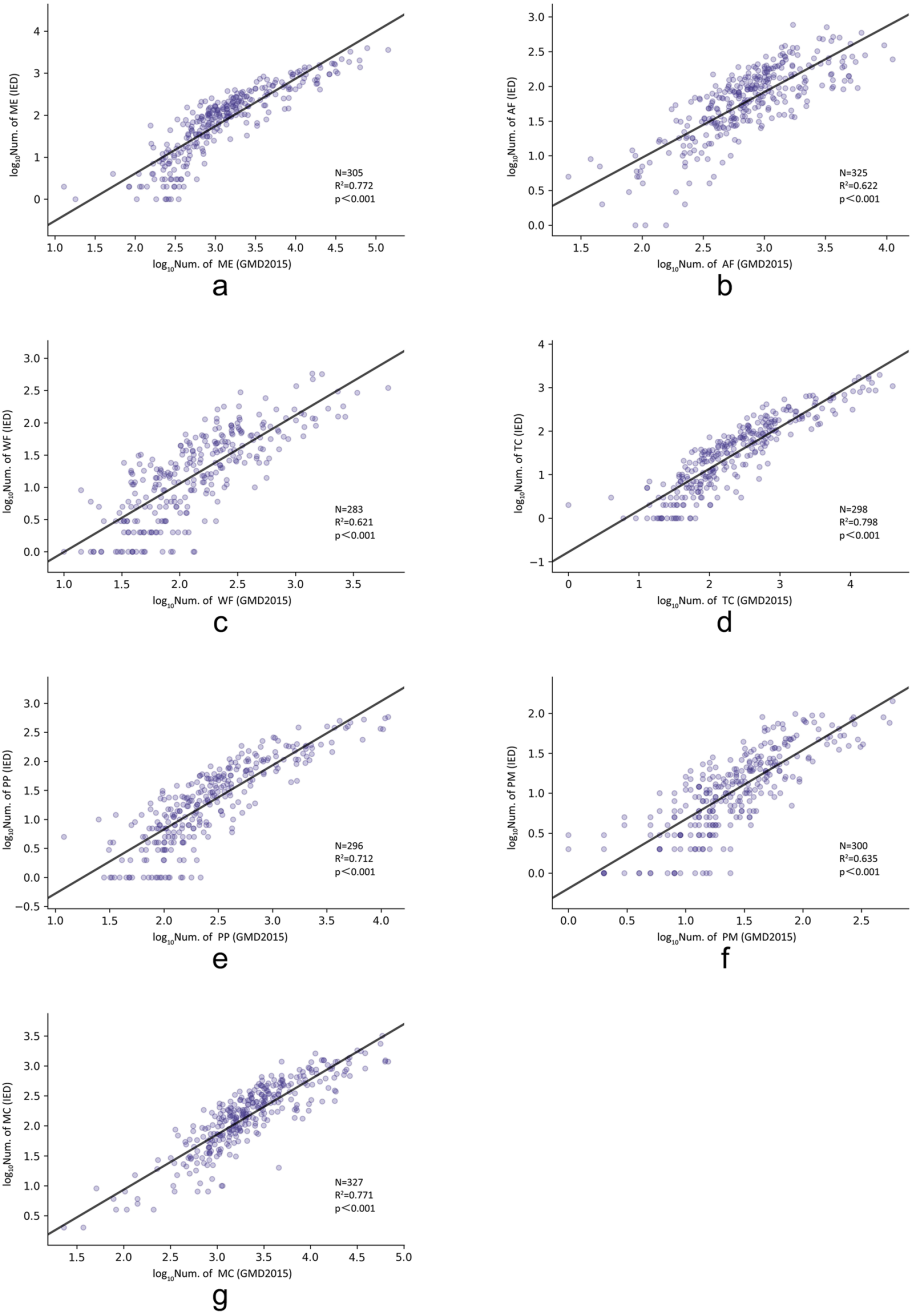
Quantity correlation analysis and verification of seven categories of manufacturing industries and industrial firm databases at the prefecture-level city scale in 2015 (**a** ME, **b** AF, **c** WF, **d** TC, **e** PP, **f** PM, **g** MC).

To verify whether the proportions of different manufacturing types in our classification results were consistent with those in the sampled data from the industrial firm database, we aggregated the number of firms into seven different types of manufacturing industries at the city level in 2015 and compared them with the IED. We present the results for the seven models in Fig. 9. In general, the proportions of manufacturing types in each city in our data were highly consistent with those sampled from the industrial firm database, with an *R*^2^ value ranging from 0.64 to 0.82. However, we found that a non-linear correlation might also appear (Fig. 8a,g). This might be explained by the fact that the number of firms sampled in the IED is low in some large cities; alternatively, the manufacturing firms in these cities might have broken into multiple branches since the IED data were collected^6^.

## Usage Notes

The GMD can be used in geographic information systems such as ArcGIS and QGIS. In GIS software, datasets can be imported as vector layers. To match with other geographic datasets (industrial park boundaries or water, air quality monitoring records^34^), users can apply spatial join capabilities in GIS software to link attributes from the GMD to other data based on spatial relationships^35^. Resampling methods can also be used if the resolution of the GMD is inconsistent with other data sources^36^.

We used 2015 and 2019, which are the two final years of China’s 12th Five-Year Plan and 13th Five-Year Plan^18^ (The data after 2020 are not representative because of the COVID-19 pandemic; consequently, research data until the end of 2019 were used), and datasets from these years provide highly representative data. If granular dynamic data need to be updated for one to two years in a specific region (such as at the province level) or in the future after 2019, our classifier can be used to process the data per these specific needs.

In addition, the names of the counties, cities, and provinces provided in this dataset are based on the administrative boundaries in 2019. The administrative divisions of the grid were determined according to the centroid of the grid. If the GMD is matched with other statistics by county, city, and province name, the effect of name changes at various scales needs to be considered. The grid cells of the GMD are the same size and location as those in the LandScan data. As the data are based on WGS84 coordinates, the spherical area of the grid cells varies among regions (the side length of the equatorial grid is approximately 1.1 km, and the side length of the Beijing grid is approximately 0.85 km).

## Data Availability

The python manufacturing classification code and machine learning samples are available at 10.6084/m9.figshare.19808407^26^.

## References

[1] Liu, D. The evolution of the world manufacturing center and China’s current situation and development trend. *Research on Development*, 76–79, 10.13483/j.cnki.kfyj.2008.05.024 (2008).

[2] Chen, Z. The shift of the world’s manufacturing center to China: trends, characteristics, conditions. *Productivity Research*, 99–101, 10.19374/j.cnki.14-1145/f.2004.06.038 (2004).

[3] Huang Q (2019). China’s manufacturing sector,industrialization and economic globalization. China Economist.

[4] Romer, Paul M (1986). Increasing returns and long-run growth. Journal of Political Economy.

[5] Krugman P (1991). Increasing returns and economic geography. Journal of Political Economy.

[6] Dong L, Yuan X, Li M, Ratti C, Liu Y (2021). A gridded establishment dataset as a proxy for economic activity in China. Sci Data.

[7] Nordhaus WD (2006). Geography and macroeconomics: New data and new findings. Proceedings of the National Academy of Sciences of the United States of America.

[8] Mao Q, Wang F, Li J, Dong S (2014). Evolving a core-periphery pattern of manufacturing industries across Chinese provinces. Journal of Geographical Sciences.

[9] Wu S, Li S (2010). An analysis of spatial distribution of manufacturing industry in China. China Soft Science.

[10] Wu J, Wei Y, Li Q, Yuan F (2018). Economic transition and changing location of manufacturing industry in China: A study of the Yangtze River Delta. Sustainability.

[11] Zhou L, Gu H, H H (2021). Evolution of China’s regional innovation structure in 2006-2018. Economic Geography.

[12] Shi M, Yang J, Long W, WEI DY (2013). Changes in geographical distribution of Chinese manufacturing sectors and its driving forces. Geographical Research.

[13] Ye H (2021). Analysis on influencing factors and spatial distribution of leisure agriculture at provincial level based on geographic big data:a case study of Zhejiang Province. China. Acta Agriculturae Zhejiangensis.

[14] Hu Y, Han Y (2019). Identification of urban functional areas based on POI data: A case study of the Guangzhou economic and technological development zone. Sustainability.

[15] Li F (2018). Big enterprise registration data imputation: Supporting spatiotemporal analysis of industries in China. Computers, Environment and Urban Systems.

[16] Li Z, Lv B (2021). Total factor productivity of Chinese industrial firms: evidence from 2007 to 2017. Applied Economics.

[17] Liu, T., Kou, F., Liu, X. & Elahi, E. Cluster Commercial Credit and Total Factor Productivity of the Manufacturing Sector. *Sustainability* **14**, 10.3390/su14063601 (2022).

[18] Shen S (2021). Research on the evolution and driving forces of the manufacturing industry during the “13th five-year plan” period in Jiangsu province of China based on natural language processing. PLoS One.

[19] Xue B, Xiao X, Li J (2020). Identification method and empirical study of urban industrial spatial relationship based on POI big data: a case of Shenyang City, China. Geography and Sustainability.

[20] Zhang H, Zhou X, Tang G, Xiong L, Dong K (2020). Mining spatial patterns of food culture in China using restaurant POI data. Transactions in GIS.

[21] Niu H, Silva EA (2021). Delineating urban functional use from points of interest data with neural network embedding: A case study in Greater London. Computers, Environment and Urban Systems.

[22] Cui Z, Huang X, He L, Zhou Z (2016). Study on urban life convenience index based on POI data. Geomatics World.

[23] Xu D, Huang Z, Lu L, Chen X, Cao F (2018). Research on spatial characteristics of urban leisure tourism based on POI mining:a case study of Nanjing city. Geography and Geo-Information Science.

[24] *Search POI* https://lbs.amap.com/api/webservice/guide/api/search (2019).

[25] *Catalogue of China Development Zone Audit Announcements* https://www.ndrc.gov.cn/fggz/lywzjw/zcfg/201803/t20180302_1047056.html?code=&state=123 (2018).

[26] Fan C (2017). figshare.

[27] Williams S, Xu W, Tan SB, Foster MJ, Chen C (2019). Ghost cities of China: Identifying urban vacancy through social media data. Cities.

[28] Zhang Z, Long Y, Chen L, Chen C (2021). Assessing personal exposure to urban greenery using wearable cameras and machine learning. Cities.

[29] Li, L. *et al*. Prediction and Diagnosis of Respiratory Disease by Combining Convolutional Neural Network and Bi-directional Long Short-Term Memory Methods. *Frontiers in Public Health* **10**, 10.3389/fpubh.2022.881234 (2022). 10.3389/fpubh.2022.881234 PMC9114643 35602136

[30] Liu, Q. *et al*. Health Communication Through News Media During the Early Stage of the COVID-19 Outbreak in China: Digital Topic Modeling Approach. *Journal of Medical Internet Research* **22**, 10.2196/19118 (2020). 10.2196/19118 PMC7189789 32302966

[31] *Administrative divisions of the People’s Republic of China* http://www.gov.cn/test/2005-06/15/content_18253.htm (2019).

[32] Limpert (2001). Log-normal distributions across the sciences: Keys and clues. BioScience.

[33] Alstott J, Bullmore E, Plenz D (2014). Powerlaw: a Python package for analysis of heavy-tailed distributions. PLoS One.

[34] He G, Wang S, Zhang B (2020). Watering down environmental regulation in China. The Quarterly Journal of Economics.

[35] *Spatial join* https://desktop.arcgis.com/en/arcmap/10.3/tools/analysis-toolbox/spatial-join.htm (2020).

[36] Lyons MB, Keith DA, Phinn SR, Mason TJ, Elith J (2018). A comparison of resampling methods for remote sensing classification and accuracy assessment. Remote Sensing of Environment.

[37] Zhang, W. & Huang, J. Evolvements of time and space in geographical concentration of China’s manufacture during 1988- 2003. *Economic Review*, 118–123, 10.19361/j.er.2007.01.021 (2007).

[38] Zhou R, Li X (2017). Evolution of spatial pattern and influencing factors of manufacturing industries in Guangdong Province. Human Geography.

[39] Zhang J, Tang G (2018). Spatial differentiation pattern of manufacturing industry in Zhejiang and its influencing factors. Scientia Geographica Sinica.

[40] Zhang X, Sun L (2012). Manufacture restructuring and main determinants in Beijing metropolitan area. Acta Geographica Sinica.

[41] Wang J (2014). Evolution of spatial pattern and influencing factors of manufacturing industries in Yangtze River Delta region. Geographical Research.

[42] Yin X, Cao F, Sun X (2019). Research on spatial agglomeration characteristics and influencing mechanism of cultural and creative industry in beijing based on POI data. Journal of Shandong Normal University(Natural Science).

[43] Chen X, Sun B (2017). Spatial structure and determinants of manufacturing employments in Shanghai metropolitan area. Human Geography.

[44] Guillain R, Le Gallo J (2010). Agglomeration and dispersion of economic activities in and around Paris: an exploratory spatial data analysis. Environment and Planning B: Planning and Design.

[45] Cao W, Zhao X, Huang X, Jin Z (2016). Influential factors of different types of industrial enterprises land intensive use in Jiangsu province. Areal Research and Development.

[46] An, T., Shi, H. & Alcorta, L. An observation and empirical study of R&D behavior of Chinese manufacturing firms:based on a survey of the manufacturing firms in Jiangsu province. *Economic Research Journal*, 21-30+56 (2006).

[47] Elgar, I. How office firms conduct their location search process? An analysis of a survey from the greater Toronto area. *International Regional Science Review*, 10.1177/0160017609331398 (2010).

[48] Chen X, Nordhaus WD (2011). Using luminosity data as a proxy for economic statistics. Proceedings of the National Academy of Sciences of the United States of America.

[49] Henderson JV, Storeygard A, Weil DN (2012). Measuring economic growth from outer space. American Economic Review.

[50] Naik N, Kominers SD, Raskar R, Glaeser EL, Hidalgo CA (2017). Computer vision uncovers predictors of physical urban change. Proceedings of the National Academy of Sciences of the United States of America.

[51] Mellander C, Lobo J, Stolarick K, Matheson Z (2015). Night-time light data: A good proxy measure for economic activity?. Plos One.

[52] Porta S (2009). Street centrality and densities of retail and services in Bologna, Italy. Environment and Planning B: Planning and Design.

[53] Zhou L (2019). How did industrial land supply respond to transitions in state strategy? An analysis of prefecture-level cities in China from 2007 to 2016. Land Use Policy.

[54] Dong H (2013). Concentration or dispersion? Location choice of commercial developers in the Portland metropolitan area, 2000–2007. Urban Geography.

[55] Dong L (2017). Measuring economic activity in China with mobile big data. Epj Data Science.

[56] Airoldi A, Janetti GB, Gambardella A, Senn L (2006). The impact of urban structure on the location of producer services. The Service Industries Journal.

[57] Llorente A, Garcia-Herranz M, Cebrian M, Moro E (2015). Social media fingerprints of unemployment. Plos One.

[58] Lv Y, Zhou L, Yao G, Zheng X (2021). Detecting the true urban polycentric pattern of Chinese cities in morphological dimensions: A multiscale analysis based on geospatial big data. Cities.

